# Supplementary material for: Does Diglossia Impact Brain Structure? Data from Swiss German Early Diglossic Speakers
Source: Brain Sci. 2024 Mar 23;14(4):304. doi: 10.3390/brainsci14040304 (PMC11048535; doi:10.3390/brainsci14040304)
Supplement: Supplementary file 1 [file brainsci-14-00304-s001.zip › brainsci-2862389-supplementary.pdf]

Supplementary Material

**Section S1**

Details on the power analysis mentioned in the text are shown below.

**Table S1.** Protocol of power analysis, G Power 3.1.9.2. *t*-test. Mean differences between two independent means (two groups). The analysis was a priori; computations required the sample size.

| | |
|---|---|
| Input: | |
| Tail (s) | =One |
| Effect size d | =0.8 |
| α err prob | =0.05 |
| Power (1-β err prob) | =0.8 |
| Allocation ratio N2/N1 | =1 |
| Output: | |
| Non centrality parameter δ | =2.5922963 |
| Critical t | =1.6838510 |
| Df | =40 |
| Sample size group 1 | =21 |
| Sample size group 2 | =21 |
| Total sample size | =42 |
| Actual power | =0.8167878 |

**Figure S1.** *t* test—mean difference between two independent means (two groups). Tail(s): one, allocation ration N2/N1 = 1, alpha err prob = 0.05, effect size d = 0.8.

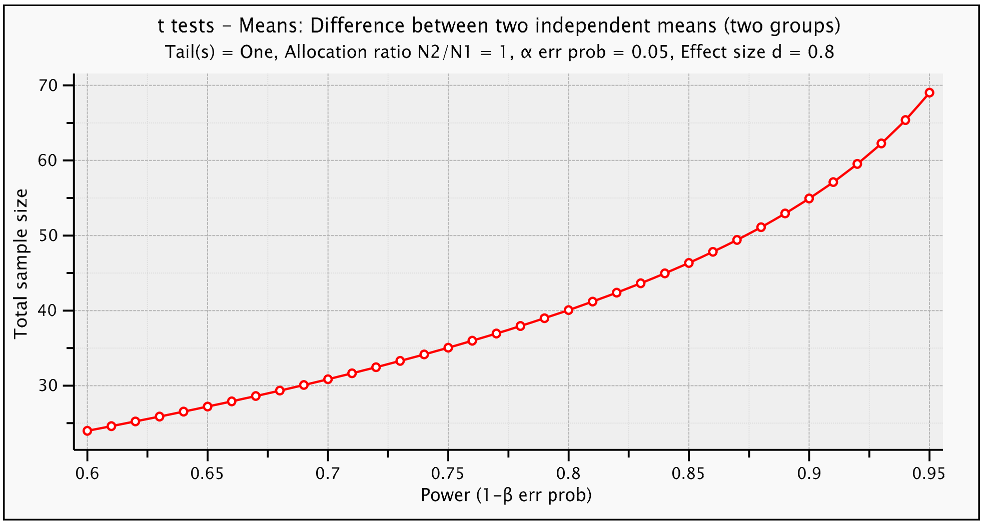


**Section S2**

Supplementary materials and raw MRI data are available in the public repository https://www.doi.org/10.5281/zenodo.10814781, accessed on the 22 January 2024 . This includes a complete pdf of the MRI acquisition protocol, all script files used during preprocessing of the data and all analysis files in order to improve the reproducibility.

**Section S3**

Supplementary analysis of matched groups in gender, number of participants and L1 and L2 exposure. The AoA of L2 was not possible to

match, as due to the school education system in Switzerland, the age varies in different regions.

**Table S2.** Analysis with matched participants. A total of 33 participants, 16 CH-GER (12 females/4 males) and 17 CH-FR (12 females/5 males). [1] imputation of one variable, [2] imputation of two variables statistics were calculated with dependent t tests.

| Variable Matched Group | L1 CH-GER | | L1 CH-FR | | |
|---|---|---|---|---|---|
| | **Mean** | **SD** | **Mean** | **SD** | ***t*-Test** |
| Age [years] | 24.63 | 8.09 | 23.29 | 2.97 | 0.54 |
| Number of spoken languages | 3.5 | 0.82 | 3.29 | 0.59 | 0.42 |
| Years of education | 15.56 [2] | 2.43 | 16.53 | 2.42 | 0.29 |
| Age of L2 acquisition [years] | 11.06 [1] | 1.06 | 8.9 | 1.22 | <0.001 |
| Current L1 exposure [% of the day] | 67.93 | 9.10 | 71.76 | 10.38 | 0.27 |
| Current L2 exposure [% of the day] | 14.13 | 11.71 | 11.06 | 8.37 | 0.40 |
| Subjective language proficiency L2 [0—none, 10—perfect] | | | | | |
| Speaking | 4.39 | 2.11 | 5.35 | 1.73 | 0.54 |
| Reading | 5.88 | 1.71 | 6.29 | 1.83 | 0.50 |
| Understanding | 6.0 | 1.93 | 6.64 | 1.69 | 0.32 |
| L2-Exposure [0–10 scale] | | | | | |
| Friends | 2.88 | 3.05 | 3.29 | 1.93 | 0.64 |
| Family | 0.81 | 1.64 | 0.47 | 0.62 | 0.44 |
| Reading | 3.5 | 2.39 | 2.65 | 1.27 | 0.22 |
| Self-study | 1.38 | 1.54 | 0.18 | 0.53 | <0.001 |
| TV | 1.31 | 1.25 | 1.06 | 1.03 | 0.53 |

*Section S3.1. Structural MRI Data with Matched Groups of a Total Number of 33 Participants*

*Whole-brain analysis*: In the t-test for the whole group, there were no statistically significant anatomic differences between the CH-GER and CH-FR groups when comparing the matter volume (VBM) and cortical thickness (SBM) of the selected group.

*Comparisons between Regions of Interest*: There was no statistical difference in the grey matter (VBM) or the cortical thickness (SBM) of the regions of interest (ROIs).

*Section S3.2. Subsection with Matched Groups of a Total Number of 33 Participants*

All five ROIs showed an identical prior probability P(M) for the alternative hypothesis compared to the null hypothesis. The error percentages were all below 0.03%.

Compared to the analysis of the whole group, the Bayesian factor indicated a higher chance that the two groups have similar volume (H0) for the left IPL and left planum temporale in the matched groups. For the other three ROIs, the results did not change, as there was still no difference in volume [68].

**Table S3.** Supplementary analysis of matching groups. Results of the group factor for Bayesian ANCOVA of the VBM with the Bayes factor ($BF_{01}$ = how much more likely a no group difference is than a difference between groups) and the error percentage [69]. Results are independent from the confounding factors (age + TIV) used as nuisance variables in the Bayesian ANCOVA. All values were directly taken from Jamovi software.

| | L1 CH-GER | | L1 CH-FR | | Bayesian ANCOVA Group Factor | |
|---|---|---|---|---|---|---|
| **Region of Interest** | **Mean** | **SD** | **Mean** | **SD** | **$BF_{01}$** | **Error %** |
| Left IPL | 0.586 | 0.0622 | 0.615 | 0.0625 | 1.52 | $9.87 \times 10^{-4}$ |
| Left planum temporale | 0.58 | 0.0913 | 0.616 | 0.0805 | 1.75 | $3.49 \times 10^{-5}$ |
| Left DLPFC | 0.605 | 0.0703 | 0.612 | 0.0516 | 2.89 | 0.027 |
| Bilateral ACC | 0.621 | 0.0792 | 0.647 | 0.0637 | 1.98 | $7.66 \times 10^{-4}$ |
| Bilateral caudate | 0.697 | 0.0814 | 0.725 | 0.0794 | 2.09 | 0.001 |
